# A Case of Septic Pulmonary Embolism Caused by Pyelonephritis With Klebsiella pneumoniae in a Patient With Poorly Controlled Type 2 Diabetes Mellitus

**DOI:** 10.7759/cureus.36098

**Published:** 2023-03-13

**Authors:** Miho Katsumata, Masaki Miura, Soichi Takizawa, Masaharu Inoue, Masahiro Masuzawa

**Affiliations:** 1 Diabetes and Endocrinology, Kameda Medical Center, Kamogawa, JPN; 2 Diabetes and Endocrinology, Yamanashi Prefectural Central Hospital, Kofu, JPN

**Keywords:** poorly controlled diabetes, urinary tract infection, septic pulmonary emboli, klebsiella pneumoniae (kp), type 2 diabetes mellitus (dm)

## Abstract

Septic pulmonary embolism (SPE) is caused by the microbe that is responsible for any clinical condition that may include urinary tract infections as in this case. We report a case of pyelonephritis with *Klebsiella pneumoniae* that led to SPE in an 80-year-old woman with poorly controlled diabetes mellitus (DM). Computed tomography (CT) revealed multiple nodules in the peripheral area of the bilateral lung and a contrast defect in the right renal vein, which was suspected to be an embolism. Blood and urine cultures revealed *Klebsiella pneumoniae* infection. These results confirmed the diagnosis of pyelonephritis and SPE. Treatment with ceftriaxone, cefazolin, and ciprofloxacin improved the patient’s condition.

## Introduction

Sepsis is characterized by a systemic inflammatory response syndrome caused by an infection and embolism means that a mass flows from elsewhere and clogs a blood vessel. Septic pulmonary embolism (SPE) is an uncommon disease in which a bacterial mass is liberated from an infected focus, causing pulmonary embolism [1,2]. Common causes of SPE with *Klebsiella pneumoniae* reported in previous studies include liver abscess, hematogenous infection, and intestinal infection; however, urinary tract infection has rarely been reported [3]. In this report, we present a case of sepsis and pyelonephritis in a patient with poorly controlled diabetes mellitus (DM) that led to SPE.

## Case presentation

An 80-year-old woman was admitted to the authors’ hospital. Approximately two weeks prior to her admission, she began to experience anorexia and gradually developed nausea and fatigue. She also felt discomfort with urination and incontinence increased. Her family found that she was unable to move, complained of intense fatigue, and was transported to the emergency room of the authors’ hospital. The weight and height of the patient were 42.4 kg and 149.7 cm, and physical examination revealed the following: blood pressure 141/80 mmHg, heart rate 104 regular beats per minute, body temperature 37.7℃, and percutaneous oxygen saturation (SpO_2_) 94%-96% breathing room air. The patient’s consciousness on the Glasgow Coma Scale was E3V4M6 as usual; however, the patient was exhausted due to intense fatigue and tenderness on palpation of the right costovertebral angle. The patient’s medical history included type 2 diabetes mellitus (T2DM), hypertension, symptomatic epilepsy, dementia, and osteoporosis. All the conditions were treated with oral medication. The surgical history included endoscopic submucosal dissection for gastric cancer at the age of 70 years. The social history indicated that the patient lived alone, and her Activities of Daily Living were full. However, the patient’s husband died approximately one year before admission, and her cognitive function declined rapidly. The patient’s medication adherence was poor because there was a large amount of leftover medicine on varying days, indicating that the patient had not taken her medication correctly. Laboratory investigations (Tables 1, 2) revealed a white blood cell count of 18,600/μL, serum C-reactive protein level of 25.371 mg/dL, a hemoglobin A1C level of 10.7%, and positive urinalysis results for uric glucose, blood, white blood cells, and nitrite.* Klebsiella pneumoniae* was detected in both blood and urine cultures, and all four bottles of blood cultures were positive in eight hours and the urine culture was positive in 16 hours (Table 3). The patient with poorly controlled T2DM was diagnosed with bacteremia triggered by a urinary tract infection.

**Table 1:** Blood test on admission WBC, white blood cell; RBC, red blood cell; Hb, hemoglobin; MCV, mean corpuscular volume; MCH, mean corpuscular hemoglobin; MCHC, mean corpuscular hemoglobin concentration; Plt, platelet; TP, total protein; Alb, albumin; BUN, blood urea nitrogen; Cr, creatinine; AST, aspartate aminotransferase; ALT, alanine aminotransferase; LDH, lactate dehydrogenase; ALP, alkaline phosphatase; Na, sodium; K, potassium; Cl, chlorine; Ca, calcium; BS, blood sugar; CRP, C-reactive protein; HbA1c, hemoglobin A1c; eGFR, estimated glomerular filtration rate; GAD, glutamic acid decarboxylase.

Variable	Reference Range, Adults	On Admission
WBC	3,900-8,800/μL	18,600/μL
RBC	380-560×10^4^/μL	411×10^4^/μL
Hb	13.4-17.5 g/dL	12.6 g/dL
MCV	83.8-103.6 fL	90.9 fL
MCH	28.3-35.3 pg	30.7 pg
MCHC	31.6%-35.9%	33.80%
Plt	13.9-37.3×10^4^/μL	22.7×10^4^/μL
TP	6.6-8.1 g/dL	6.1 g/dL
Alb	4.1-5.1 g/dL	2.1 g/dL
BUN	8.0-20.0 mg/dL	13.7 mg/dL
Cr	0.65-1.07 mg/dL	0.5 mg/dL
AST	13-30 U/L	15 U/L
ALT	10-42 U/L	19 U/L
LDH	124-222 U/L	288 U/L
ALP	33-113 U/L	126 U/L
Na	138.0-145.0 mmol/L	133.1 mmol/L
K	3.6-4.8 mmol/L	3.9 mmol/L
Cl	101.0-108.0 mmol/L	93.9 mmol/L
Ca	8.8-10.1 mg/dL	8.5 mg/dL
BS	73-109 mg/dL	374.0 mg/dL
CRP	0.000-0.140 mg/dL	25.371 mg/dL
HbA1c	4.9%-6.0%	10.7%
eGFR	60 mL/min/1.73m^2^	87.0 mL/min/1.73m^2^
Anti-GAD antibody	<5.0 U/mL	<5.0 U/mL

**Table 2:** Urinalysis on admission WBC: white blood cell.

Variable	Reference Range, Adults	On Admission
Specific gravity	1.000-1.030	1.010
pH	5.0-7.0 (pH)	6.0 (pH)
Protein	Negative (mg/dL)	1+ (mg/dL)
Blood	Negative (mg/dL)	2+ (mg/dL)
Glucose	Negative (mg/dL)	4+ (mg/dL)
Ketone	Negative (mg/dL)	3+ (mg/dL)
Urobilinogen	Negative (mg/dL)	± (mg/dL)
Nitrite	Negative (mg/dL)	2+ (mg/dL)
Leukocyte	Negative (WBCs/µL)	2+ (mg/dL)

**Table 3:** Cultures on admission CFU, colony-forming unit.

Variable	Species	Comments
Blood	Klebsiella pneumoniae	All four bottles positive
Urine	Klebsiella pneumoniae	10^6 ^CFU/mL

**Figure 1:**
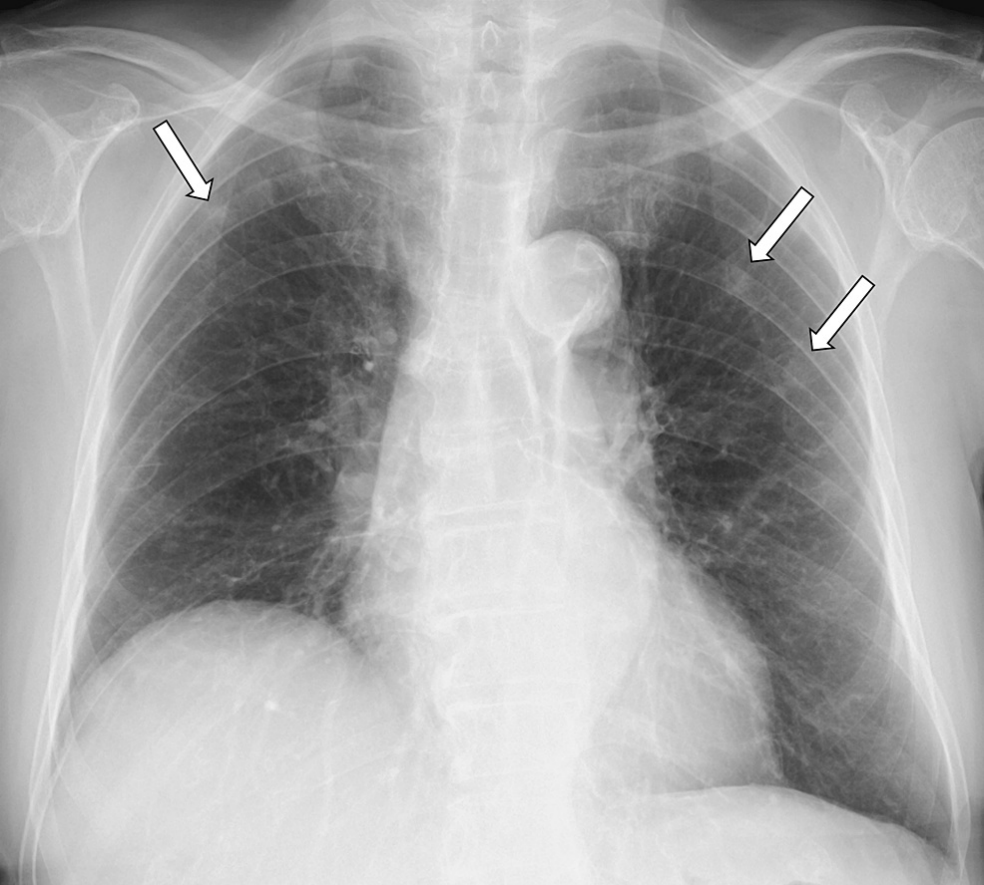
Image of chest radiograph on admission Multiple nodules in the upper bilateral lobe. The arrows indicate pulmonary nodules.

Transthoracic echocardiography revealed normal wall motion, no vegetation, and no severe valvular disease. Chest radiography (Figure 1) revealed multiple nodules in the upper bilateral lobes, and no cardiac enlargement, consolidation, or pleural effusion was observed.

**Figure 2:**
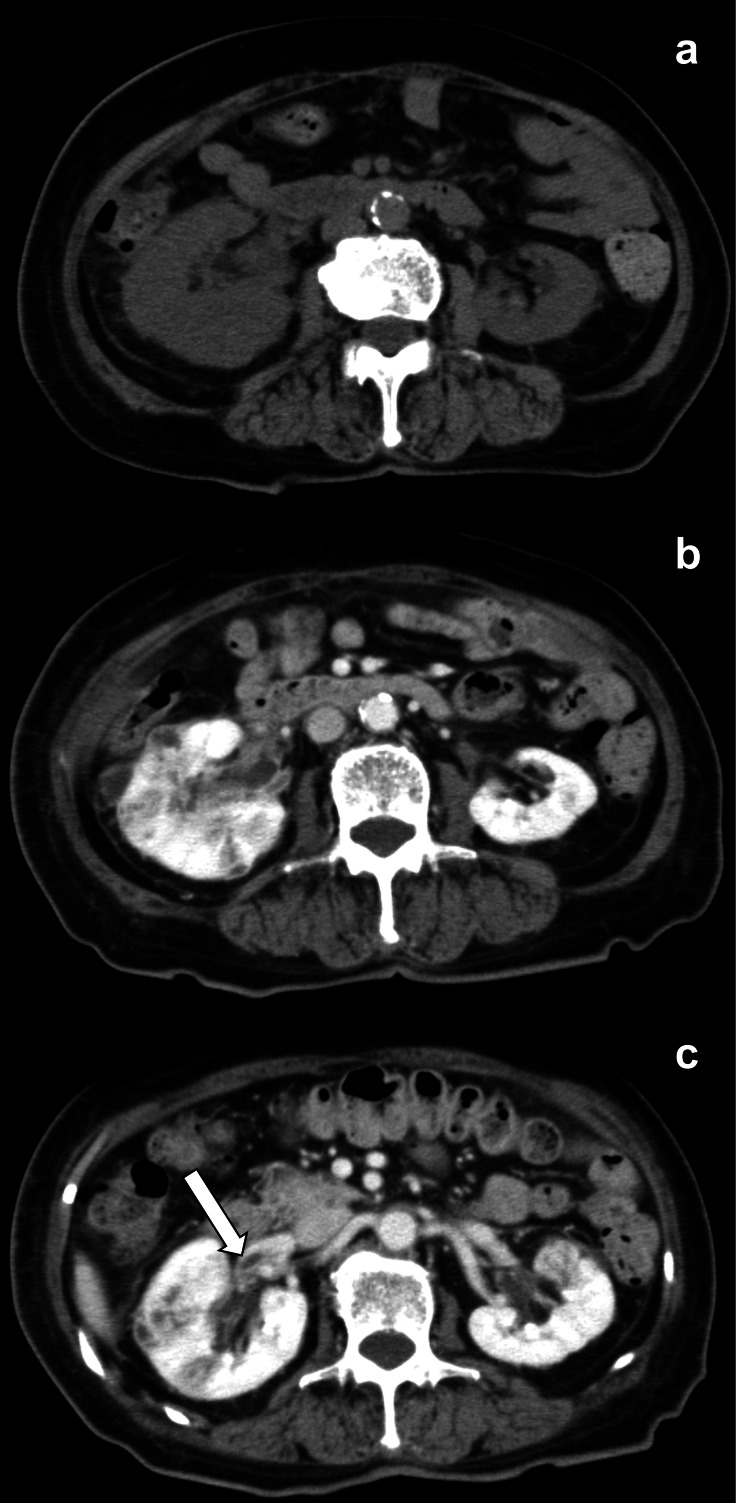
Images of abdominal CT Bilateral renal swelling and perinephric standing are also observed (a). Poor contrast enhancements in bilateral renal parenchyma are observed (b). Suspected bacterial mass in the right vein. The arrow indicates a suspected bacterial mass (c).

**Figure 3:**
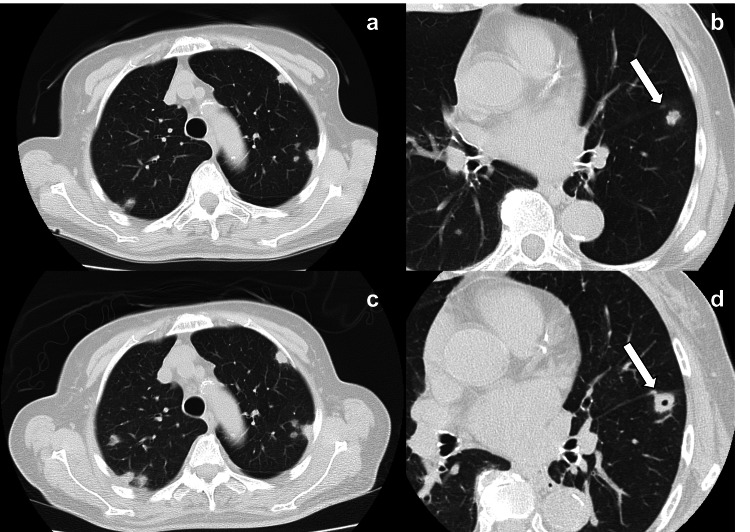
Images of chest CT Multiple nodules are observed in the peripheral area on admission (a, b). Multiple nodules are enlarged and cavities appeared in some of them on day 4 (c, d). The arrows indicate a nodule with enlargement and a cavity.

Plain abdominal computed tomography (CT) (Figure 2a) revealed bilateral renal swelling and perinephric standing with no ureteral stones or urinary tract obstruction. Plain chest CT (Figures 3a, 3b) revealed multiple nodules in the peripheral area. Pulmonary metastasis of a malignant tumor was suspected, and contrast-enhanced CT was performed on day 4. Contrast-enhanced CT (Figure 2b) showed poorly contrasted areas in both kidneys. Moreover, a contrast defect (Figure 2c) was revealed in the right renal vein, and a bacterial mass was suspected. Multiple nodules in the bilateral lobe enlarged in three days, and cavities appeared in some of them (Figures 3c, 3d).

Liver abscesses were not observed. A mass of about 18 mm in the left kidney was observed, and renal cell carcinoma was suspected; however, it was determined to be too small to be related to multiple nodules. Consequently, there were no observations suggesting a suspected malignant tumor to be the primary focus of the nodules. Other laboratory results confirmed pyelonephritis in poorly controlled T2DM that led to SPE. First, ceftriaxone 2 g per day intravenous (IV) was initiated, and after the confirmation of bacterial species and antimicrobial susceptibility (Table 4), it was de-escalated to cefazolin 6 g per day IV. The treatment was switched to ciprofloxacin 800 mg per day per oral on day 15. *Klebsiella pneumoniae* in this case was not extended-spectrum β-lactamase-producing.

**Table 4:** The antimicrobial susceptibility on admission MIC, Minimal Inhibitory Concentration; S, susceptible; I, intermediate; R, resistant; CLSI, Clinical and Laboratory Standard Institute; ABPC, ampicillin; PIPC, piperacillin; CVA/AM, clavulanic acid/amoxicillin; IPM, imipenem; MEPM, meropenem; CEZ, cefazolin; CFPM, cefepime; CMZ, cefmetazole; CTM, cefotiam; CTX, cefotaxime; AMK, amikacin; GM, gentamicin; CPFX, ciprofloxacin; ST, sulfamethoxazole/trimethoprim.

Drug Name	MIC	CLSI Decision
ABPC	≥32	R
PIPC	32	I
CVA/AM	≤2	S
IPM	0.5	S
MEPM	≤0.25	S
CEZ	≤4	S
CFPM	≤1	S
CMZ	≤1	S
CTM	≤8	S
CTX	≤1	S
AMK	≤2	S
GM	≤1	S
CPFX	≤0.25	S
ST	≤20	S

**Figure 4:**
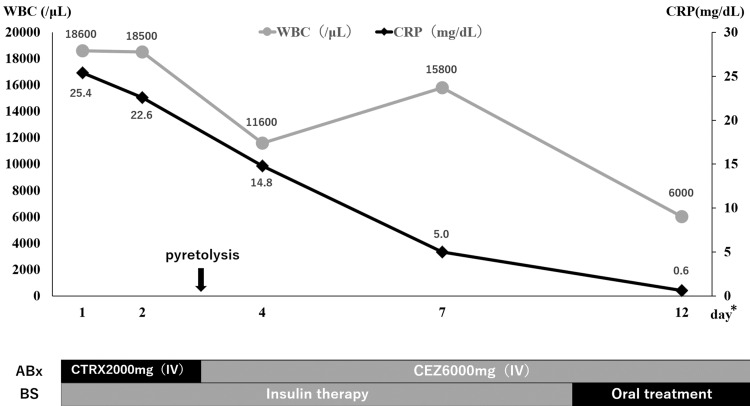
Process of treatment and inflammatory markers The symbol * indicates days of hospitalization. The treatment was initiated with ceftriaxone 2 g per day. After confirming bacterial species and antimicrobial susceptibility, it was de-escalated to cefazolin 6 g per day IV on day 3. Insulin was also initiated to control blood glucose and then switched to oral medication. After treatment initiation, the patient's temperature resolved, and inflammatory markers rapidly decreased. WBC, white blood cell; CRP, C-reactive protein; ABx, antibiotics; BS, blood sugar; CTRX, ceftriaxone; CEZ, cefazolin; IV, intravenous.

Finally, antibiotics were intravenously administered for two weeks and orally for two weeks, for a total of four weeks. Regarding blood glucose management, insulin was initiated on admission but discontinued after resuming oral medication on day 10. Blood glucose level was in good control ranging from 110 to 160 mg/dL. The temperature increased to 38°C on day 2 but resolved on day 3 and inflammatory markers reduced rapidly (Figure 4).

**Figure 5:**
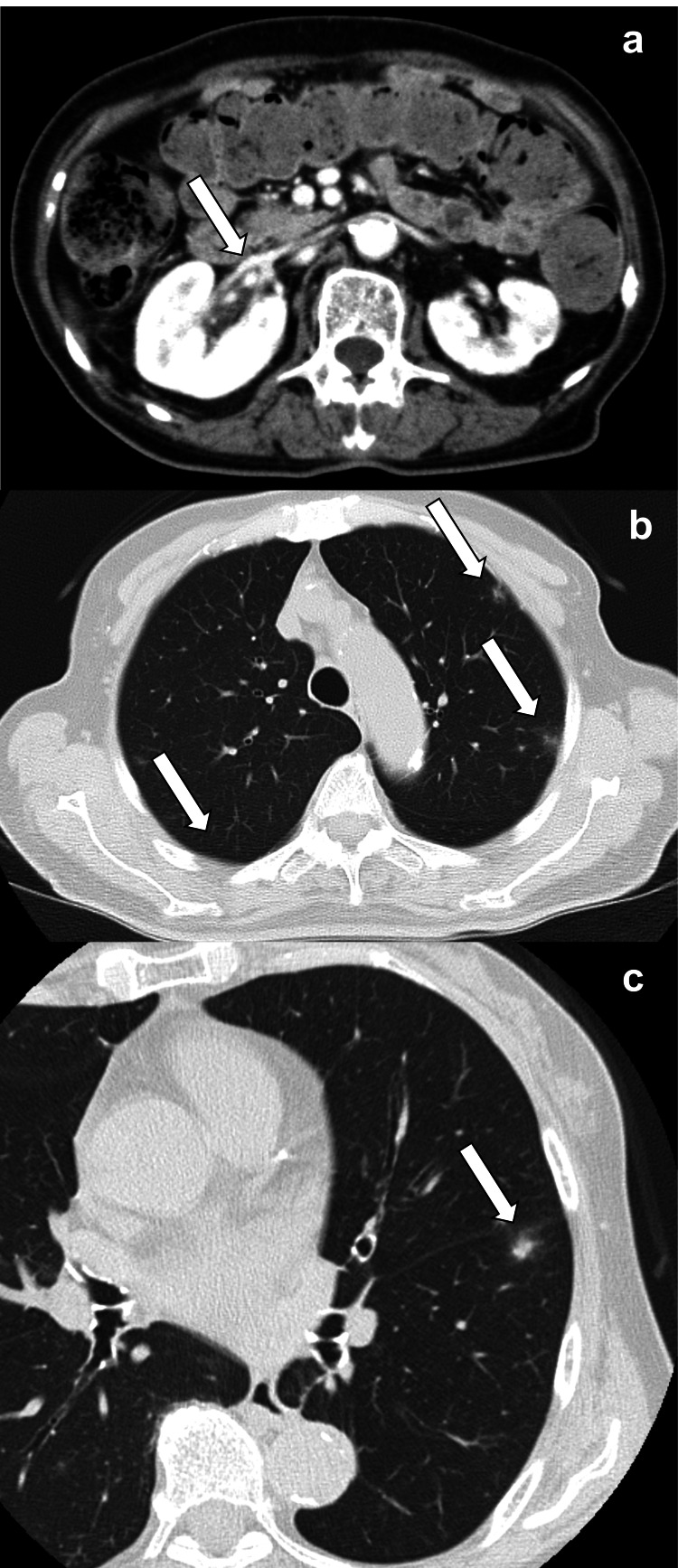
One-month follow-up CT after treatment initiation Bilateral renal swelling improved, and poor contrast enhancement regressed, and the arrow indicates the point at which a suspected bacterial mass was detected on day 4 (a). Multiple nodules and cavities regressed or disappeared. The arrows indicate nodules and cavities with regression and disappearance (b, c).

The nutrition condition was good, and rehabilitation was initiated on day 5 to prevent disuse syndrome. The patient’s general condition improved, and she was transferred to a rehabilitation hospital on day 18. On contrast-enhanced CT (Figure 5a) one month after the initiation of treatment, bilateral renal swelling had improved remarkably, and poor contrast areas were reduced. All multiple nodules in the bilateral lobe had diminished or reduced (Figures 5b, 5c). The patient in this case report provided informed consent.

## Discussion

SPE is an uncommon non-thrombotic disease in which a bacterial mass is liberated from an infected focus via venous circulation, causing pulmonary embolism [1,2]. Image features of SPE reveal multiple nodules 0.5-3.5 cm in size, feeding vessel signs consisting of a distinct vessel leading directly to nodules or masses, and varying degrees of cavitation predominantly in the peripheral and lower lung fields [4,5]. In this case, the observations were consistent with SPE, indicating multiple peripheral nodules and some cavities. The cause of cavity formation is considered to be the interruption of blood flow by the embolism, leading to necrosis and infection of the embolized tissue [6]. In a systematic review of 168 cases, the common primary infective foci of SPE were drug addiction (44 cases), intervascular indwelling catheters (21 cases), infective endocarditis (20 cases), and liver abscesses (15 cases), and blood cultures grew commonly in methicillin-sensitive *Staphylococcus aureus* (MSSA) in 48 cases, methicillin-resistant *Staphylococcus aureus* (MRSA) in 27 cases, and* Fusobacteria* in 11 cases. Only three patients had urinary tract infections, and *Klebsiella pneumoniae *grew in 11 cases [7]. Moreover, in SPE with *Klebsiella pneumoniae*, the common primary infective focus of SPE in 33 cases was a liver abscess (26 cases), hematogenous infection (8 cases), and intestinal infection (three cases); however, urinary tract infection was absent [3]. In this case, *Klebsiella pneumoniae* was detected in both blood and urine cultures. *Klebsiella pneumoniae* is a gram-negative rod-shaped bacterium belonging to the* Enterobacteriaceae *family. Although it is very rare for SPE to develop purely from pyelonephritis with *Klebsiella pneumoniae* as in this case, it is well known that renal cell carcinoma can form tumor thrombus and metastasize to the lungs via the venous circulation rather than through lymphatic spread [8]; therefore, it can be explained that a “metastatic infection” develops from the pathway of renal vein, inferior vena cava, and right ventricular system to pulmonary artery.

DM increases the risk of urinary tract infections. A meta-analysis of 22 articles from 1966 to 2007 reported that both male and female patients with DM had a higher frequency of asymptomatic bacteriuria [9]. In an evaluation of urinary tract infections in patients over 18 years of age and between January 1 and December 31, 2010, both cystitis and pyelonephritis were more common in patients diagnosed with T2DM than in those without T2DM during one-year follow-up [10]. Moreover, the impact of an environment with hyperglycemia on *Klebsiella pneumoniae* infections was evaluated. High glucose concentrations surrounding the bacteria decrease the phagocytosis and bactericidal activity of neutrophils, leading to an increased synthesis of bacterial polysaccharide capsules [11,12]. In this case, pulmonary metastasis of a malignant tumor was suspected first; however, poorly controlled T2DM, CT images, association with pyelonephritis, the same result of *Klebsiella pneumoniae* in blood and urine cultures, and no observation of malignant tumors confirmed the diagnosis of SPE. The embolism suspected in the right renal vein (Figure 2c) suggested that the circulation to the lungs resulted in SPE. Hyperviscosity in DM seems to be strongly related to hyperglycemia and is influenced by the quality of diabetic control [13]. This suggests hypercoagulability and the promotion of embolization. This patient had poorly controlled DM with a hemoglobin A1c (HbA1c) level of 10.7% on admission, which is considered to increase the risk of SPE.

## Conclusions

While it is common for SPE to develop from a liver abscess, it is very rare for SPE to develop from pyelonephritis. We experienced a case of pyelonephritis complicated by SPE, which was treated with antibiotic therapy. In cases of pyelonephritis from *Klebsiella pneumoniae* in poorly controlled type 2 diabetic patients, SPE should also be considered.
